# Remembering Mumps

**DOI:** 10.1371/journal.ppat.1004791

**Published:** 2015-05-07

**Authors:** Donald R. Latner, Carole J. Hickman

**Affiliations:** Division of Viral Diseases, National Center for Immunization and Respiratory Diseases, Centers for Disease Control and Prevention, Atlanta, Georgia, United States of America; University of Florida, UNITED STATES

## What Is Mumps?

The mumps virus belongs to the family of paramyxoviruses. It has a single-strand, nonsegmented, negative-sense RNA genome and is spread by the respiratory route. Following a 12–25-day incubation period, infection frequently causes the classic symptom of mumps: painfully swollen parotid salivary glands (parotitis). Some complications of infection include hearing loss, orchitis, oophoritis, mastitis, and pancreatitis. Mumps may also result in aseptic meningitis and, infrequently, encephalitis (5%–10% and <0.5% of unvaccinated cases, respectively) [6]. Importantly it has been estimated that as many as 30% of infections in unvaccinated individuals may be asymptomatic [7].

## What Do We Know about the Immune Response to Mumps?

The reasons why symptomatic mumps infections occur among vaccinated individuals are not clear because there are no definitive correlates of protective immunity for mumps. It is assumed that neutralizing antibody is essential for protection, but repeated attempts to define a protective threshold titer have been inconclusive [8]. Some evidence suggests that memory T lymphocytes are probably necessary to confer protection, but they are likely not sufficient [9]. By several measures, the immune response to mumps virus (both wild type and vaccine) seems inherently weak. The average mumps in vitro plaque reduction neutralization titer is low (typically ≤1:256) even after wild-type infection. In addition, the predominant antibody response appears to be directed to the nucleoprotein, which is a non-neutralizing target [10]. Finally, some reports indicate that the frequency of mumps-specific memory B lymphocytes is very low [11,12]. This could be due to poor antigenicity and low abundance of viral proteins during infection or possibly due to an inadequate T cell response.

## Has the Virus Changed?

It is logical to suspect antigen drift as a possible explanation for breakthrough mumps infections among previously vaccinated populations. Although there are 12 recognized genotypes of mumps, there is only one serotype [13], meaning that antibody generated in response to infection with one strain of the virus can recognize the most genetically divergent strains. Interestingly, however, some reports indicate that there is often 2-to-16-fold variation in the amount of sera required from any given individual to neutralize genetically diverse mumps strains in vitro [14,15]. The reasons for this are not clear, but a possible cause is subtle variation in the neutralizing epitopes. Considering these observations, it has been postulated that an individual with very low levels of neutralizing antibody may become susceptible to some wild-type strains of the virus as his or her neutralizing antibody titer wanes.

There is evidence that mumps antibody may be boosted in vaccinated individuals by asymptomatic wild-type infection [16]. As endemic mumps virtually disappeared in the US, a consequent lack of natural boosting may have contributed to a reduction in population immunity to a level that is capable of sustaining transmission in some settings. Notably, recent outbreaks in the US have generally occurred in circumstances that promote a high frequency and intensity of contact, such as college dormitories, boarding schools, and youth summer camps, and spread of the virus beyond these settings into surrounding communities has been limited [17].

## How Protective Is Mumps Vaccine?

The effectiveness of two doses of MMR vaccine is different for each virus component [2]. In contrast to the measles and rubella vaccines, which are ≥95% effective, reports of mumps vaccine effectiveness vary and range from 79%–95%, with a median of 88% [18]. Although it is imperfect, the protection afforded by mumps vaccination is effective, valuable, and important. As mentioned, high vaccination coverage has nearly eliminated endemic disease in the US and has limited spread of the virus to settings of high-intensity exposure. Furthermore, as compared to the prevaccine era, there has been a reduction in the frequency of complications among vaccinated individuals, which indicates there are important measures of protection that should not be overlooked [17]. Finally, there is no evidence of immune escape, indicating that vaccination should induce antibody that is capable of neutralizing wild-type virus.

Because of the lack of a well-defined correlate of immunity, it is not currently possible to predict with confidence whether or not someone who has been vaccinated is susceptible or protected. If a vaccinated individual does not have detectable mumps antibody, they may likely be susceptible, but this is not a forgone conclusion. The amount and specificity of antibody or other components of immunity that might be required for protection are simply not known.

Limited data are available regarding the effectiveness of a third dose of mumps vaccine [10]. During recent outbreaks when a third dose was given as a control measure, there was a reduction in disease incidence [19,20]. However, it is not clear from these reports if the outcome was a direct result of third-dose vaccination or if the reduction was simply the natural decline in disease incidence due to the late timing of the intervention in the course of the outbreak. In addition, limited antibody boosting was observed following third-dose vaccination, except in individuals who had extremely low (or no) mumps antibody [10].

## Do We Need a New Mumps Vaccine and How Can We Make a Better One?

High expectations exist for the effectiveness of modern vaccines, in part due to the tremendous historic success of vaccination against viruses such as smallpox, polio, measles, rubella, and also mumps. It is important to remember, however, that not all pathogens are equivalent. There are significant fundamental differences in the ways they are transmitted, the pathologies they cause, and their interactions with the host immune system. Some of these differences may be reflected in the effectiveness of the respective vaccines and the longevity of the immune response to each.

It is reported that wild-type mumps (re)infections can occur more than once in the same individual [21]. Based on this observation—that not even wild-type mumps infection necessarily confers lifelong immunity—an important question is, what level of protection can be reasonably expected of any mumps vaccine? A more effective mumps vaccine that provides lifelong immunity is certainly desirable [22], but before scientifically grounded improvements can be made, it will be essential to better understand which parameters of immunity are required for protection and why the immune response to mumps is characteristically weak. Good animal models that accurately and consistently mimic the pathology and immune response to mumps virus infection in humans are lacking, although rhesus macaques tend to mirror the response the best and may be useful for future studies [23].
